# A qualitative study of midwives’ challenges to support transmen during childbirth: A short report

**DOI:** 10.18332/ejm/116410

**Published:** 2020-02-07

**Authors:** Margareta Johansson, Andrea A. Wirén, Damali Ssempasa, Michael Wells

**Affiliations:** 1 Uppsala University, Akademiska sjukhuset, Uppsala, Sweden; 2 Karolinska Institutet Medical University, Stockholm, Sweden

**Keywords:** intrapartum care, lack of knowledge, midwives, transmen

## Abstract

**INTRODUCTION:**

Individuals who are transgender often want a family and want to be validated as fathers, but may lack support in the reproductive health field. The aim of this qualitative study was to explore midwives’ perceptions regarding caring for transmen during labour and birth.

**METHODS:**

Five midwives were recruited from Stockholm-area hospitals, with interviews lasting 17 minutes on average. Qualitative content analysis using an inductive approach was used.

**RESULTS:**

Our findings describe the challenges midwives face when caring for transmen in childbirth, including a lack of knowledge, confusion on working with transgender, how to provide individualized support, and the complexity of childbirth.

**CONCLUSIONS:**

The midwives faced challenges when caring for transmen in childbirth. Since midwives lacked knowledge regarding best practices to support transmen, they wanted to receive more education on how to care for transmen during birth.

## INTRODUCTION

Individuals who are transgender often want a family^1^, and to be validated as fathers/parents^2^. However, transmen might experience transphobia and lack support from within the reproductive health field^2^, affecting their childbirth experience^3^. The aim of this study was to explore midwives’ perceptions regarding caring for transmen during labour and birth.

## METHODS

### Study design

A descriptive qualitative design was applied for the study^4^.

### Setting

Three Swedish hospitals in the capital of Sweden were chosen as study sites.

### Participants

This qualitative study included five midwives. The inclusion criteria for participating were: 1) fluency in Swedish, and 2) be a midwife who currently works in a labour ward.

### Measures and variables

Individual audio recorded semi-structural interviews were carried out. As an introduction for each interview, the interviewer made the statement: ‘Think about an ordinary day at the labour ward. You are told to take care of a person in labour who is a man, and his partner’. A follow-up statement was then made: ‘Tell me in detail about your thoughts regarding this scenario’. Interviews lasted between 12–21 minutes, with an average of 17 minutes.

### Data analysis

Qualitative content analysis using an inductive approach by Graneheim and Lundman^4^ was used. Interviews were transcribed verbatim, and three authors checked the transcriptions for quality assurance. The original transcripts were read and then re-read to give a deeper understanding of its content. Same statements that related to the same central meaning (meaning units) were identified and labeled (coded). Each content that shared a commonality was thereafter sorted into four different categories that described the study theme: ‘Challenging to provide labour support to transmen’^4^.

### Ethical procedures

No formal ethical approval was sought since supporting pregnant transmen is part of their professional care. However, department heads and hospital managers approved the study plan, and an information letter was provided to and informed consent was signed by the participants. In addition, no identifiable information was collected or reported.

## RESULTS

All midwives were female and aged 28–56 years (mean 43 years). They had intrapartum care experience ranging 1–24 years (mean 9 years). No midwife had ever worked directly with a pregnant transmen. The study theme was explored and is described by four categories (Table 1).

**Table 1:** Explored theme and categories

*Categories*	*Theme*
Perceived lack of knowledge	Challenging to provide labour support to transmen
Confusion on working with gender	
Wanting to provide quality intrapartum care	
The complexity of childbirth	

### Perceived lack of knowledge

A lack of knowledge regarding transmen in childbirth was described.

*‘I don’t think there’s so much knowledge, because when people were told about this interview, they said, But what, how are they [the babies] going to come out, through the Dick or?’*. (Midwife 4)

Midwives stated that they had limited personal experiences with transmen. As such, they did not know about the effects of hormone treatments on birth and if this would affect the birth, and how they would react.

*‘I think it’s okay to feel a bit uncomfortable with things you don’t know so much about’*. (Midwife 1)

*‘I haven’t seen any guidelines, nor attended any lecture arranged by my work place, and I don’t think any special knowledge exist’*. (Midwife 2)

Direct experiences, they noted, would lead to greater knowledge and higher self-efficacy in caring and supporting transmen.

### Confusion on working with gender

Midwives thought that transmen disliked acknowledging their biological genitals, and that by transforming into a man (e.g. via hormones), while still having female organs, could be psychologically and emotionally conflicting.

*‘If there is an aversion and a feeling of disconnection with that part, not wanting to acknowledge that part of the body, so when you are supposed to give birth to a baby, it may be a brutal reminder of your sex’*. (Midwife 2)

Midwives thought that if transmen found it difficult to handle labour because of the focus on vaginal examinations, then the focus should change to be on the baby. Furthermore, midwives were concerned with how to document an infants’ birth if the parents did not want to assign a gender to their infant.

### Wanting to provide quality intrapartum care

Midwives felt that it was important to offer individual intrapartum support regardless of gender identity. Respect, dignity and humility were highlighted as important aspects of support.

*‘It`s still a human being who is going to give birth to a baby, so what does it matter [to be a transman] in that context?’*. (Midwife 2)

Midwives were unsure of how to address transmen, but noted they wanted to address them appropriately so they did not feel excluded, invisible or distrusting of the health care services.

*‘I wonder, how should I address, and how will I feel by calling someone Johnny, “Come on Johnny, push!” maybe this will be absurd and unaccustomed’*. (Midwife 2)

*‘Oh, what if I say mom or something… that I define incorrectly’*. (Midwife 3)

Midwives believed continuity of care was preferable, so transmen would not need to re-explain their status.

*‘I think many would feel good about knowing the midwife who will be present during labour and birth, especially if having low trust in health care or low self-confidence’*. (Midwife 2)

Other suggestions for better support included having transmen write a personal letter and/or a birth plan into their antenatal medical journal, as well as inform the hospital before arriving about their gender identity. During the labour process, the midwives regarded an open dialogue as important to provide individual support.

*‘We need to meet on an individual level and acknowledge just this person or what this couple needs in order for them to feel well and secure’*. (Midwife 3)

*‘I will give same kind of childbirth support; I make no difference’*. (Midwife 4)

### The complexity of childbirth

The midwives struggled articulating who gives birth: a person, human, woman, transman, or man. Therefore, they noted that the organs, such as the uterus and vagina, might give birth. They thought it was odd to see a man giving birth, but felt that all individuals had the right to give birth regardless of their gender identity.

*‘Although this man may have a penis and no breasts but it’s still the uterus and vagina that give birth to the baby’*. (Midwife 1)

*‘I`ve been thinking about the visual image of a person who looks like a man, is addressed as a man but still has a pregnant belly and is going to give birth to a baby through a vagina’*. (Midwife 2)

*‘Childbirth may be experienced as hard because there is such a focus on the uterus, vaginal examinations and such’*. (Midwife 3)

While the midwives thought that other midwives might not be as open-minded about transmen in childbirth, they were positive about transmen giving birth, and noted that while it could be exciting or fun, it may also be uncomfortable and nerve wracking.

*‘Anyway, it`s a fantastic and big experience in life to have a baby. It will change you forever, regardless of being a female or a transman giving birth’*. (Midwife 3)

## DISCUSSION

Our findings describe the challenges midwives face when caring for transmen in childbirth, including a lack of knowledge, confusion on working with transgender, transmen’s physical functions and what kind of individualized support they need. While midwives wanted to provide high quality intrapartum care, they needed more knowledge regarding how to care for transmen during childbirth. Midwives thought that transmen could write a personal letter and/or a birth plan into their antenatal medical journal so that they could provide more personalized care. While patients can and should self-promote their own well-being, midwives in the current study suggested few options of how they should change their behavior to better support transmen beyond receiving more education.

Transmen often experience internal conflict between their sense of self and dominant social norms that define a pregnant person as a woman and gestational parent as a mother^5^. However, midwives are supposed to promote, protect and support sexual and reproductive health, and respect cultural diversity and human dignity^3^. Since the participants noted that they lacked experience with and knowledge about how to best support transmen in childbirth, it might be difficult for them to adhere to these policies; therefore, a greater emphasis on how to best support transmen in labour is warranted.

### Limitations

Limitations to the study include a small sample size and relatively short interviews. In addition, all midwives were positive about supporting transmen in their birth experience, but none had actual previous experience with transmen in childbirth. Therefore, future research is needed to explore the transferability of these findings.

Since individualized childbirth support is important for transmen’s emotional, psychological, and physical health, midwives might benefit from better education and professional development support regarding best practices for these parents.

## CONCLUSIONS

The midwives faced challenges when caring for transmen in childbirth. To provide high quality care, midwives need to increase their knowledge and skills regarding supporting transmen, especially during labor and birth.
